# Engineering Cell Membrane‐Based Nanotherapeutics to Target Inflammation

**DOI:** 10.1002/advs.201900605

**Published:** 2019-05-22

**Authors:** Huize Yan, Dan Shao, Yeh‐Hsing Lao, Mingqiang Li, Hanze Hu, Kam W. Leong

**Affiliations:** ^1^ Department of Biomedical Engineering Columbia University New York NY 10027 USA; ^2^ Guangdong Provincial Key Laboratory of Liver Disease The Third Affiliated Hospital of Sun Yat‐sen University Guangzhou Guangdong 510630 China; ^3^ Institutes of Life Sciences School of Biomedical Science and Engineering and National Engineering Research Center for Tissue Restoration and Reconstruction South China University of Technology Guangzhou International Campus Guangzhou Guangdong 510006 China; ^4^ Department of System Biology Columbia University Medical Center New York NY 10032 USA

**Keywords:** biomimetic nanomedicine, cell membrane engineering, inflammation, inflammatory microenvironment, targeted drug delivery

## Abstract

Inflammation is ubiquitous in the body, triggering desirable immune response to defend against dangerous signals or instigating undesirable damage to cells and tissues to cause disease. Nanomedicine holds exciting potential in modulating inflammation. In particular, cell membranes derived from cells involved in the inflammatory process may be used to coat nanotherapeutics for effective targeted delivery to inflammatory tissues. Herein, the recent progress of rationally engineering cell membrane‐based nanotherapeutics for inflammation therapy is highlighted, and the challenges and opportunities presented in realizing the full potential of cell‐membrane coating in targeting and manipulating the inflammatory microenvironment are discussed.

## 1 Introduction

Inflammation is the process by which a harmful stimuli‐triggered protective response initiates a coordinated cascade of complex regulatory networks to defend pathogenic infection, tissue damage or autoimmune signals.^1^ It is increasingly appreciated that chronic or uncontrolled inflammation drives the development and progression of various devastating diseases, including cancer, obesity, type 2 diabetes, atherosclerosis, ischemic disease, systemic inflammatory response syndrome, and even autoimmune disease.^2^ Although numerous attempts have been made to overcome these complex diseases, the therapeutic response remains unsatisfactory partly due to disease heterogeneity.^3^ In spite of the diseases mentioned above have diverse phenotypic consequences, they share several common denominators in the dysregulation of immune homeostasis.^4^ In such a scenario, the inflammatory response aims to restore immune homeostasis, especially in the inflammatory microenvironment, which contains invasive pathogens and damaged tissues in addition to infiltrated immune cells.^5^ It has become evident that multiple immune cells play a central role in shaping the inflammatory microenvironment, balancing immune homeostasis.^6^ In response to the primary inflammatory stimulus, activated immune cells are preferentially homed to the site of the inflammatory microenvironment, and produce pro‐ or anti‐inflammatory mediators, which could modulate the inflammatory responses to homeostatic status.^7^ With increasing understanding of the connection between inflammation and homeostasis, a holistic consideration of the inflammatory microenvironment over the course of disease progression offers unique opportunities for designing more specific and efficacious diagnostic as well as therapeutic agents to target and regulate inflammation. For instance, recent studies have provided new and exciting insight into immunotherapies, from protein delivery to gene therapy, on how to modulate immune homeostasis to achieve remission of chronic inflammation.^8^ In addition to these immunomodulation therapies, great strides have been made to engineer autologous immune cells to precisely correct a complex disease phenotype to achieve optimal outcomes.^9^ However, the main drawback associated with these immune therapies is inadequate efficacy and unwanted adverse effects, which might be attributed to the poor localization at the appropriate site of action after systemic administration or the clearance and destruction by inflammatory cells after local administration.^10^ Thus, developing advanced delivery strategies to improve the modulation of the inflammatory microenvironment could facilitate the treatment of acute or chronic inflammation.

The advent of nanomedicine marks an unparalleled opportunity to develop mainstream solutions for a wide variety of healthcare predicaments.^11^ With synergistic progress in nanotechnology, biomaterial, and immunology, there has been an exponential increase in the design of nanoplatforms for inflammation diagnosis, therapy, monitoring, and the balance of immune homeostasis.^12^ Inflammation‐specific nanoplatform is typically constructed with responsiveness to the inflammatory microenvironment after the nanocarriers have reached the target site.^13^ It may mean that the nanocarrier composed of synthetic materials or natural biopolymers can degrade to release the cargo in response to an inflammatory stimulus such as depressed pH or elevated level of reactive oxygen species, or the nanocarrier itself can possess intrinsic anti‐inflammatory properties.^14^ To facilitate navigation to the inflammatory microenvironment, much effort has focused on decorating the nanoplatforms to evade systemic clearance by the reticuloendothelial system (RES) or to enhance tissue biodistribution by targeting inflammatory cell receptors.^15^ However, there remains much room for improvement.^16^ Harnessing the advances in nanomedicine and our understanding of inflammatory microenvironment would help the development of bioinspired nanomedicines, where design cues for personalized inflammatory modulation can be taken from nature. For instance, we can learn from how hemocytes and stem cells home to inflamed tissue and drive homeostasis, and design biomimetic strategies to target inflammatory disease.^17^ One approach is to fabricate inflammation‐related cell‐derived or mimicking nanoparticles as drug delivery systems to prolong their circulation times and to achieve specific and sensitive inflammatory microenvironment targeting. This can be done by coating the nanoparticles with cell membranes.

Cell membrane‐based nanotherapeutics has emerged as a promising approach to facilitate delivery of therapeutic agents via integrating the biomimetic features of cell membranes with the functional versatility of nanomaterials.^18^ This facile top‐down approach is characterized by “cloaking” synthetic nanocarriers with a layer of the natural cell membrane, which helps to overcome some of the drawbacks associated with nanoparticles fabricated by conventional self‐assembly such as poor colloidal stability and nonspecific tissue accumulation.^19^ Sophisticated engineering of these cell membrane characteristics facilitates nanocarriers to favorably accumulate in the target tissue.^20^ Cell membrane‐cloaked nanoparticles could inherently reproduce the biological properties of the source cells from which their membrane is derived, aiming to achieve a wide range of functions such as prolonged circulation and disease‐relevant targeting.^21^ Promising results have emerged recently in molecular imaging, drug delivery and immune therapy.^22^

**Figure 1:**
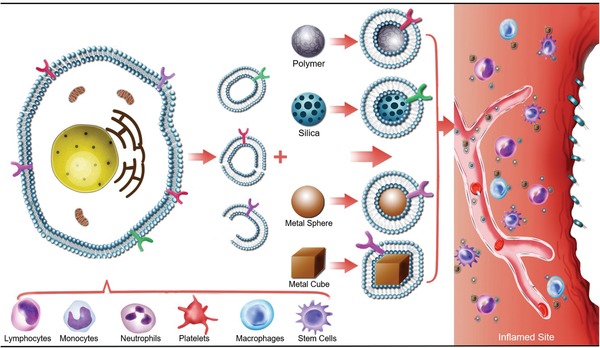
Cell membrane‐based nanotherapeutics for inflammation. After rational engineering, a variety of types of cells (lymphocytes, monocytes, neutrophils, platelets, macrophages, stem cells, etc.) have been employed as sources of coating materials to prepare cell membrane‐based nanotherapeutics. The applicable nanoparticle cores include organic nanoparticles (polymers), inorganic nanoparticles (silica nanoparticle, metal nanoparticle), hybrid nanoparticles (MOFs), and so on. Its shape and structure can be chosen based on the target sites and applications. By leveraging the advantages of inherent properties from the source cells, these biomimetic nanotherapeutics bestow a wide range of functions in targeting, monitoring, and regulating inflammatory diseases.

Diverging from existing reviews, we focus only on the recent progress in exploiting engineered cell membrane‐based nanoplatforms for targeting inflammatory disease (summarized in **Figure** **1**). We begin with a comprehensive overview of state‐of‐the‐art cell membrane engineering, extraction, and cloaking technologies, specifically intelligent cell membrane engineering for specific inflammation targeting and modulation. Next, we offer insights on how to select inflammation‐related cells and how these cells interact with the inflammatory microenvironment. We also highlight the recent advances in materials and medical science that have allowed for the precise manipulation of cell membrane‐based nanoplatforms for targeting, monitoring, and regulating inflammatory diseases. Finally, we discuss opportunities and challenges for the future clinical translation of cell membrane‐based therapeutics.

## 2 Cell Membrane Engineering, Extraction, and Cloaking Technologies

The preparation of cell membrane‐coated nanoparticles involves three crucial steps: engineering of the cell membrane, extraction of membrane‐derived vesicles, and the fusion of synthetic nanocarriers with cell membrane‐derived vesicles.^23^

### 2.1 Strategies to Engineer Cell Membrane Properties

**Figure 2:**
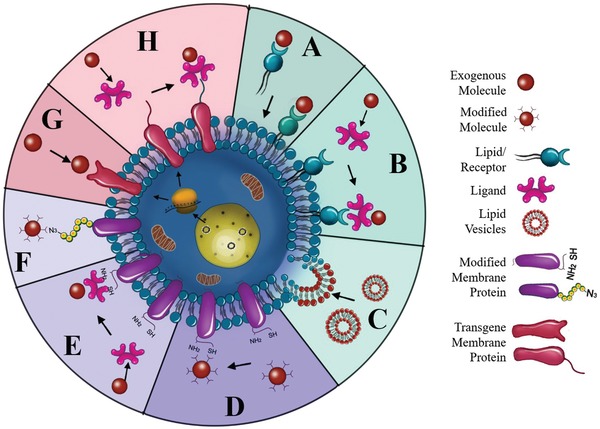
Strategies for cell membrane engineering. Summary of the main modification strategies including A–C) physical, D–E) chemical, and G,H) biological engineering of the cell membrane. A) Direct insertion of lipid‐conjugated molecules into cell membranes. B) Insertion of exogenous groups/receptors into cell membranes followed by binding with ligand‐conjugated molecules. C) Fusion between molecule‐containing lipid vesicles and cell membranes. D) Direct covalent conjugation of molecules with primary amine or thiol residues on cell membranes. E) Introduction of biotin reactive groups onto cell membranes and binding with avidin‐ or streptavidin‐functionalized molecules. F) Modification of azido groups on polysaccharides of cell membranes followed by linkage with molecules. G) Directly genetic introduction of transgene protein expression on the cell membranes. H) Genetic introduction of “bio‐orthogonal” reactive groups/receptors onto cell membranes followed by binding with molecules.

The composed of organized lipids, polysaccharides, and proteins, is instrumental for cell to communicate with its microenvironment.^24^ Since cell membranes play a crucial role in many cellular phenotypes, including adhesion, migration, recruitment, and cell‐cell interactions, modifying the cell membrane would be an interesting approach to endow the membrane‐coated nanocarrier with additional properties beyond those conferred by the natural cell membrane.^25^ The three modification strategies can be roughly categorized as physical, chemical, and genetic (**Figure** **2**).

#### 2.1.1 Physical Engineering of the Cell Membrane

**Figure 3:**
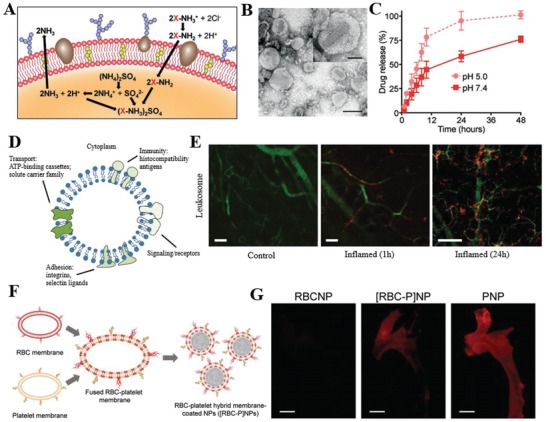
Physical strategies for cell membrane engineering. A) Concept of fusion red blood cell (RBC) membrane‐derived vesicles with cholesterol for loading of doxorubicin (Dox). B) Representative TEM image of Dox‐RBCs (scale bar 100 nm). C) Dox release profile of Dox‐RBCs at pH 5.0 or pH 7.4. Reproduced with permission.^29^ Copyright 2017, Wiley. D) Schematic representation of leukosome bilayer with multiple molecules involved in transport, signaling, immunity and adhesion. E) Fluorescence images of inflamed‐vasculature (green) targeting relative to rhodamine‐labeled leukosomes (Red). Reproduced with permission.[qv: 30b] Copyright 2016, Nature Publishing Group. F) Schematic of RBCs and platelet membrane fusion and coating PLGA nanocores to produce [RBC‐P]NPs. G) Distribution of DiD‐labeled [RBC‐P]NPs in the aortas from mice with atherosclerosis. Reproduced with permission.^32^ Copyright 2017, Wiley.

The physical engineering of cell membrane centers around the lipid structure and its membrane fluidity.^26^ Given that some glycosylphosphatidylinositol (GPI) can be naturally anchored to cell membranes through the lipid‐lipid interaction, GPI‐fused proteins can be engineered to attach to cell membranes.^27^ Inspired by this observation, lipid‐conjugated molecules with targeted or therapeutic properties can be attached to cell membranes by inserting the hydrophobic lipid portion into the outer leaflet of lipid bilayers (Figure 2A). Exogenous receptors exemplified by lipid mimics can also be inserted into cell membranes to engineer cells to bind to ligands such as probes, therapeutics, and biomacromolecules with a high affinity (Figure 2B).^28^ Additionally, liposomes were introduced to fuse with cell membrane‐derived vesicles, facilitating the encapsulation and controlled release of small molecules (Figure 2C). To this end, red blood cell (RBC) membrane‐derived vesicles were first fused with cholesterol to fabricate a liposomal carrier with pH gradient maintenance, which exhibited encapsulation of the chemotherapeutic doxorubicin and antibiotic vancomycin with pH‐dependent drug release behavior (**Figure** **3**A–C).^29^ These drug‐loaded, cholesterol‐fused cell membrane vesicles were able to outperform the corresponding free drugs in both a murine model of breast cancer and a methicillin‐resistant *S. aureus* (MRSA) skin infection model, with fewer concerns regarding safety and immunogenicity. The accumulation of leukocytes (neutrophils, lymphocytes, etc.) in tissue sites would contribute to various diseases (see Sections 3.1.1 and 3.1.2). Since the homing property of leukocytes is crucial for targeting the inflammatory microenvironment, leukocyte membranes have been isolated to design nanoplatforms enabled with leukocyte‐like inflammation targeting properties.[qv: 17a,30] In this regard, a similar strategy of camouflaging nanoparticles was undertaken through the incorporation of liposomes with enriched leukocyte membrane protein to form proteolipid vesicles, referred to as leukosomes (Figure 3D,E).[qv: 30b] This novel strategy allowed the transfer of key transmembrane proteins, including CD45 and CD47 with a long circulation property as well as macrophage‐1 antigen (Mac‐1), lymphocyte function‐associated antigen (LFA)‐1 and P‐selectin glycoprotein ligand‐1 (PSGL‐1) with superior adhesion of the inflamed endothelium to the surface of leukosomes to overcome the inflamed vascular barrier.[qv: 30c] Importantly, leukosomes retained the versatility and physicochemical properties of typical liposomal formulations, preferentially targeted inflamed vasculature, enabled the selective and effective delivery of dexamethasone to inflamed tissues, and escaped the mononuclear phagocytic system in a localized model of inflammation.[qv: 30b,31] The versatility of this leukocyte‐lipid fusion strategy may facilitate the next generation of leukosomes for the treatment of a broad range of disorders that have few therapeutic alternatives but share a similar inflammatory microenvironment.[qv: 30a] Although the abovementioned strategies show promise on targeting and therapy, they are not able to completely combine the intrinsic features of multiple cell membranes. To address this issue, a new type of cell membrane engineering strategy has emerged by fusing the cell membranes of two different types of cells. A recent study showed that the fused membrane from RBC and platelet when coated on a poly(lactic‐*co*‐glycolic acid) (PLGA) core could carry the properties of both source cells (Figure 3F,G).^32^ The obtained [RBC‐P]NPs maintained the platelet‐based atherosclerosis‐targeting characteristics, as well as the RBC platelet‐based extended blood circulation time, thereby outperforming their single membrane counterparts. We propose that this strategy might potentially be used to integrate functions across multiple inflammation targets. Collectively, physical engineering strategies are convenient and compatible with other cell membrane modification schemes to fabricate engineered cell membranes for multiple applications. However, the instability of the inserted molecules might limit the effectiveness of the physical approach.

#### 2.1.2 Chemical Engineering of the Cell Membrane

**Figure 4:**
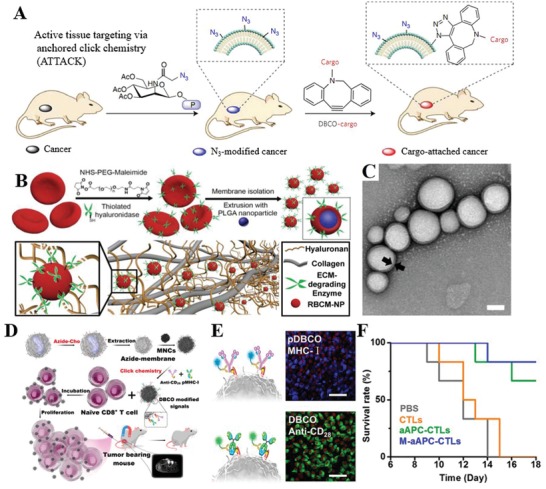
Chemical strategies for cell membrane engineering. A) Schematic illustration of ATTACK (active tissue targeting via anchored click chemistry) technology. Reproduced with permission.^42^ Copyright 2017, Nature Publishing Group. B) Schematic illustration of the fabrication of PH20‐RBCM‐NPs and their diffusion in the extracellular matrix. C) Representative TEM image of PH20‐RBCM‐NPs, scale bar represents 50 nm. Reproduced with permission.^43^ Copyright 2016, Ivyspring International Publisher. D) Schematic illustration of the fabrication of biomimetic artificial antigen‐presenting cells (aAPC) for T‐cell‐based anticancer therapy. E) Fluorescence images of J774A.1 macrophages sequentially exposed to DBCO‐antiCD28/DBCO‐pMHC‐I, scale bar represent 50 µm. F) Survival rate of mice treated with M‐aAPC‐CTLs. Reproduced with permission.^44^ Copyright 2017, American Chemical Society.

The chemical engineering strategies would mainly be based on the primary amine and thiol residues of membrane‐associated proteins and the hydroxyl residues of polysaccharides.^33^ These groups can be used as active sites for various covalent conjugation schemes. Functional molecules containing carboxyl groups can be easily conjugated to the amino residue of cell membranes through a versatile 1‐ethyl‐3‐(3‐dimethylaminopropyl) (EDC)‐mediated amidation reaction (Figure 2D).^34^ Although EDC itself causes some cytotoxic effects by activating the carboxyl groups from cell organelles and membranes, EDC‐treated antigen‐presenting cells exhibited unique tolerogenic responses when interacting with T cells.^35^ To achieve faster and safer chemical engineering of the cell membrane, functional molecules with preactivated carboxyl groups can be directly linked with the cell membrane. For instance, functional molecules including polymers, nucleic acids, and proteins can be preactivated with N‐hydroxysuccinimide (NHS) or N‐hydroxysulfosuccinimide sodium salt (NHS‐sulfo) for direct conjugation to the cell membrane.[qv: 34a,36] In addition to the well‐known EDC‐meditated bioconjugation method, maleimide‐functionalized molecules can be used to conjugate to the high level of thiol residues on hemocytes, including red blood cells, monocytes, leukocytes, and hematopoietic stem cells (HSCs).^37^ Although these strategies provide sustained pseudoautocrine stimulation to engineered cells for targeted drug delivery, the cells might lose their functions and viability. Functional molecules lacking a carboxyl moiety cannot be conjugated to the cell membrane via a one‐step conjugation; a multistep reaction would be needed, such as the introduction of biotin onto the cell membrane (Figure 2E). Specifically, avidin‐ or streptavidin‐functionalized molecules or nanoparticles can be linked through the strong biotin‐avidin recognition.^38^ Recently, a novel bifunctional linker named succinimidyl‐[(N‐maleimidopropionamido)‐diethyleneglycol] ester (NHS‐PEG_2_‐maleimide) with cell impermeable properties was designed to conjugate amine‐rich cell membranes and thiol‐activated peptides with NHS and maleimide groups, respectively.^39^ These peptide‐engineered stem cells not only showed firm adhesion and rolling on E‐selectin under physiological shear stresses but also maintained their viability, proliferation, or multipotency. Following these studies, murine splenocytes were reacted with NHS‐PEG‐N_3_ to obtain azide groups on the surface, and transforming growth factor beta 1 (TGF‐β1) protein was modified with methyl‐2‐(diphenylphos‐phino) terephthalate (MDT) using NHS‐PEG‐MDT.^40^ Then, MDT‐activated TGF‐β1 was immobilized onto the azide‐functionalized antigen‐presenting cell (APC) surface through a mild biorthogonal reaction, providing ease in translating this strategy to innumerable biomaterial platforms to modulate the transplant environment. In addition, an alternative to introduce chemically reactive groups onto the cell surface is to convert the hydroxyl residues of the abundant polysaccharides on cell membrane to the aldehyde groups with the aid of sodium cyanoborohydride.^41^ Nanoparticles with a hydrazine moiety could be conjugated to the aldehyde groups to form stable secondary amine linkages through the Mannich reaction (Figure 2F). Previous attempts have been made to selectively label target cells with azido groups through tetraacetyl‐N‐azidoacetylmannosamine (Ac4ManAz)‐mediated click chemistry, which subsequently allowed the enhanced tumor accumulation of dibenzocyclooctyne (DBCO)‐doxorubicin conjugate via click chemistry to provide targeted cancer therapy against several selected tumor types in mice (**Figure** **4**A).^42^ This so‐called active tissue targeting via anchored click chemistry (AttAcK) strategy might be expandable to the design of other sugar derivatives for selective azide cell labeling in various inflammatory diseases along with inflammation‐specific triggers.

With the aid of the abovementioned NHS‐PEG_2_‐maleimide biofunctional linker, RBCs were first engineered with a human recombinant hyaluronidase (rHuPH20), and then RBC membrane‐coated nanoparticles (RBCM‐NPs) were fabricated to possess the hyaluronidase activity as well as ultralong blood circulation time (Figure 4B,C).^43^ The RBCM‐NPs exhibited efficient diffusion in the peri‐cellular hyaluronic acid matrix of cells, offering the potential to target and image inflammatory atherosclerotic plaques with abundant hyaluronic acid. In addition to engineering RBCs, the leukocyte membrane was pre‐engineered with azide‐modified lipids and conjugated with dibenzocyclooctyne‐activated T‐cell‐stimulated antibody (anti‐CD28) or peptide (pMHC‐I) through mild and highly efficient copper‐free click chemistry (Figure 4D–F).^44^ Then, the magnetic nanoclusters (MNCs) were cloaked with engineered membrane‐derived vesicles to form artificial APCs, leading to more efficient T‐cell expansion and increased cytotoxicity. By taking advantage of the magnetic properties of MNCs and the targeting properties of the cell membrane, these artificial APCs could be delivered in a targeted fashion by magnetic control, and their accumulation could be monitored through magnetic resonance imaging (MRI). This strategy also has great promise for adoptive T‐cell‐based anticancer immunotherapy and provides insight into inflammation targeting and imaging. To date, the state‐of‐the‐art bio‐orthogonal chemistry offers a powerful tool in the tailored engineering of living cell membranes.^45^ These chemical strategies are convenient in endowing cells with new functions while preserving their biological competence. However, one drawback is the lack of reaction specificity, which might damage the bioactivity of the native proteins. These drawbacks might be circumvented by complementing the chemical engineering strategy with the physical and biological engineering approaches.

#### 2.1.3 Biological Engineering of the Cell Membrane

**Figure 5:**
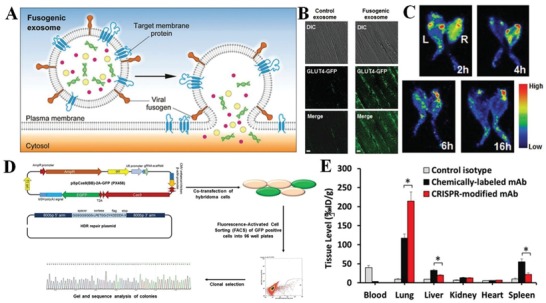
Biological strategies for cell membrane engineering. A) Schematic illustration of cell membrane engineering by fusogenic exosomes. B) Representative fluorescence images of GLUT4‐GFP in mouse skeletal TA muscle fibers at 4 h after exosome treatment. C) Representative images of PET scans of glucose uptake obtained from the femur muscles of BALB/c nude mice of each group (left, control exosomes; right, fusogenic exosomes). Reproduced with permission.^53^ Copyright 2017, Wiley. D) Schematic illustration of the CRISPR/Cas9 genome editing approach of hybridoma cells for site‐specific modification of antibodies. E) Biodistribution of ^111^In‐labeled anti‐ICAM mAb at 30 min after administration. Reproduced with permission.^58^ Copyright 2018, Nature Publishing Group.

Genetic engineering offers selective introduction of desired proteins or peptides on cell membranes through transfection or transduction via nonviral or viral vectors, respectively (Figure 2G).^46^ It is widely used to modify cell membranes with fused motifs for targeting and therapeutic applications.^47^ Mesenchymal stem cells (MSCs) exhibit an inherent ability to home to inflammatory environment and exert immunosuppressive functions; therefore, MSCs have been genetically engineered to over‐express membrane proteins such as C‐X‐C motif receptor 4 (CXCR4), C‐C chemokine receptor type‐1 (CCR‐1), PSGL‐1, and tetrasaccharide sialyl Lewis X (SLeX) to enhance their homing to inflammatory environments.^48^ For instance, intercellular adhesion molecule (ICAM)‐1 antibody‐engineered MSCs exhibited higher adhesion to activated endothelial cells in vitro and enhanced homing in a model of inflammatory bowel disease (IBD).^49^ Advances in genetic engineering combined with an improved understanding of immune cell recognition have led to the introduction of chimeric antigen receptors (CARs) on T cells, which redirect antigen specificity and enhance their function in adoptive immunotherapy.^50^ In addition to membrane proteins, nonmembrane proteins or peptides can also be co‐expressed on the cell membrane with vesicle membrane proteins, including glycosylphosphatidylinositol (GPI), the C1C2 domain of lactadherin, and platelet‐derived growth factor (PDGF).^51^ For instance, cell‐based vesicles harboring GPI‐anchored hyaluronidase (PH20) have been designed to degrade hyaluronan, thereby augmenting nanoparticle penetration and drug diffusion.^52^ These chemotherapeutic‐loaded engineered nanoplatforms exhibited higher activity in cancer therapy than the previously studied recombinant PH20 proteins, which could be applied to targeting and regulating inflammation. In a similar report, a fascinating approach was developed by producing fusogenic cell vesicles harboring VSV‐G (as the fusogen), and tetraspanin CD63 fused with green fluorescent protein (CD63‐GFP, as a reporter for exosomal membrane proteins) (**Figure** **5**A–C).^53^ These fusogenic exosomes could efficiently deliver GFP‐fused CD63 (CD63‐GFP) or glucose transporter‐4 (GLUT4‐GFP) to the recipient cell membrane to allow the increased glucose uptake of recipient cells, highlighting the potential of these bioinspired nanoplatforms for targeted delivery of membrane proteins. Rather than direct chemical engineering, genetic engineering can also be used to introduce “bio‐orthogonal” reactive groups into the cell membrane surface, creating sites for the selective engineering of cells through a combination of traditional molecular biology and exogenous chemical methods (Figure 2H).^54^ Cell membrane‐anchored peptides with specific amino acid sequences can be selected as the targeted acceptor of enzymes, which catalyze the conjugation of acceptor peptides and functional molecules.^55^ For instance, thioester acyl residues of the LPETGG motif cleaved by Sortase A were able to realize the linkage of N‐terminal glycine‐functionalized peptides on the cell membrane.^56^ This bio‐orthogonal strategy was well established to prepare protein–protein or protein–drug conjugates and provides a promising approach to engineer multifunctional cell membranes to target inflammation. To facilitate the introduction of acceptor peptides, the clustered regularly interspaced short palindromic repeats (CRISPR)/CRISPR‐associated protein 9 (Cas9) system has been used, which enables the insertion of genes at predefined regions of the genome.^57^ In a recent study, the LPXTG motif was genetically expressed on RBC membranes through the CRISPR/Cas9 system, and the immunodominant peptide that contains a suitably exposed N‐terminal glycine could be covalently linked to the LPXTG motif with the aid of Sortase A (Figure 5D,E).^58^ These disease‐associated autoantigen engineered RBCs induced antigen‐specific tolerance, therefore alleviating and exhibiting prophylactic and therapeutic efficacy in experimental autoimmune encephalomyelitis (EAE). Overall, although viral or nonviral methods may cause some uncontrolled toxicity or immunogenecity concerns of engineered cells, biological engineering strategies with improved specificity could be combined with other chemical or physical strategies to achieve precise cell membrane engineering, especially for the modulation of inflammatory microenvironments.

### 2.2 Strategies to Prepare Cell Membrane‐Based Vesicles

Cell membranes are considered homologous phospholipids embedded in functional surface proteins or polysaccharides.^59^ After cell membrane engineering, the extraction of cell membranes can typically be divided into three steps: cell breaking, membrane collection, and vesicle formation. For nucleus‐free cells, including RBCs, platelets and bacteria, the cells are first isolated through several centrifugation‐based approaches.^60^ Then, RBCs or platelets could be lysed with either a repeated freeze‐thaw process or hypotonic incubation.^61^ Gram‐negative bacterial membranes would be replaced by secreted outer membrane vesicles (OMVs) because it difficult to extract bacterial membranes due to the peptidoglycan proteins of cell walls.^62^ The cell membrane fragments are then purified with centrifugation and subsequently extruded serially through polycarbonate membranes with gradient pores to prepare the final vesicles at the nanoscale. The extraction of membranes from cancer cells, leukocytes, and stem cells is more complicated than the extraction from nucleus‐free cells.^63^ Large number of cells (more than ten million) must be harvested from culture systems, blood, or tissue samples. After purification and enrichment, cells incubated with hypotonic lysis solutions are broken with homogenization or sonication, or a combination thereof.^64^ The cell membrane fragments would then be washed, purified, and extruded following the steps mentioned above. The entire extraction step should proceed at 4 °C and as gently as possible, and protease inhibitors and endotoxin free solution are highly recommended for the storage of cell membrane‐based vesicles at −80 °C to achieve long‐term maintenance of membrane protein function.^65^ In addition to the traditional strategy, a novel method was reported recently.^66^ The cells were serially filtered through polycarbonate membranes with gradient micrometer pores to directly obtain the membrane fragments. Then, these fragments were washed, purified and extruded to small vesicles. This rapid, convenient, and economical approach might expedite the preparation of cell membrane‐derived vesicles to form bioinspired nanoplatforms.

### 2.3 Strategies to Cloak Cell Membranes on Synthetic Nanoparticles

Cell membrane‐cloaked nanoparticles can be fabricated using three main strategies: extrusion, sonication, and microfluidics. Initial work has relied on the co‐extrusion of synthetic nanoparticles with cell membrane‐derived vesicles through a nanoporous membrane.^61^ Inspired by the formation of lipid‐enveloped hybrid nanoparticles, researchers increasingly use sonication to generate disruptive forces for the spontaneous fusion of synthetic nanoparticles and cell membrane‐derived vesicles to form a core‐shell nanostructure.^67^ New approach is also seen in using electropolation to promote the fusion of the nanoparticles and cell membrane fragments when the mixture flows through a microfluidic channel.^68^ The size, shape, surface charge, and the ratio of core nanoparticles to coating materials control the efficiency of cell membrane coating.^69^ Notably, the negatively charged nanoparticle core is necessary for successful cloaking.^61^ It is not only a key factor in fabricating the core‐shell structure but also crucial for the formation of right‐side‐out structures. However, the negatively charged nanoparticle cores could induce significant aggregation due to the collapse of the fluidic lipid bilayer and impede the local arrangement necessary for lipid coverage through electrostatic interactions. Beyond these cell membrane‐derived and vesicle‐based strategies, in situ production of membrane‐coated nanoparticles was reported regardless of their chemistry surface or shape.^70^ This unique strategy was achieved by incubating living cells with nanoparticles and collecting the nanoparticle‐loaded vesicles. Furthermore, the systematic characterizations of the resulting cell membrane‐cloaked nanoparticles for their physiochemical and biological functions are necessary before biological experiments.^19^ In this regard, the efficiency of the cell membrane coating can be determined through morphological observations, size, and charge change, permeability, protein composition, and cellular colocalization.

## 3 Engineering Cell Membranes for Inflammation

Inflammation has been found in almost every pathological condition. The origin and development of infectious and noninfectious diseases such as atherosclerosis, arthritis, type 1 diabetes, systemic lupus erythematosus, and inflammatory bowel disease (IBD) are more or less associated with a certain series of inflammatory cascade responses. Therefore, inflammation‐targeted therapy has become a burgeoning concept guiding clinical treatment, especially in chronic and unresolved conditions. Deeper understanding of how the inflammatory microenvironment works and how it is linked with cell homing and cell communication would facilitate the engineering of cell membranes for inflammation. In this section, several inflammation‐related cells, including hematocytes and stem cells, together with their recruiting and targeting behavior will be first considered. It will be followed by the discussion of applying these cell membranes to nanotherapeutics for inflammation monitoring and therapy.

### 3.1 Inflammation‐Related Cell Types and Their Targeting Mechanisms

**Figure 6:**
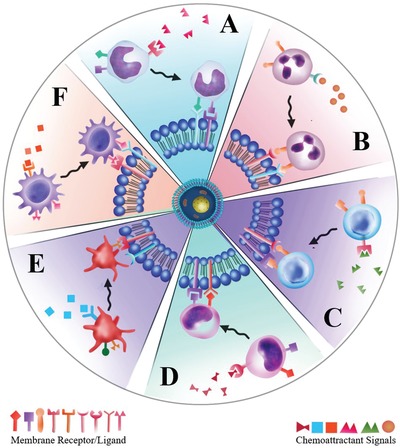
Schematic illustration of inflammatory‐related cell targeting process. Once cells such as A) monocytes, B) neutrophils, C) macrophages, D) lymphocytes, E) platelets, and F) stem cells in the circulating blood stream receive chemoattractant signals (e.g., chemokines, pathogen products, and cytokines) from inflamed sites, they will be activated and migrate to the site, and activate cell–cell interactions with local cells.

Inflammation‐related cells, such as neutrophils, macrophages, lymphocytes, platelets, and stem cells, undergo a two‐step recruiting process that is precisely controlled in a temporal and spatial manner. Resting cells in the circulation become activated by pathogen products, cytokines or chemokines, and subsequently migrate to the site of infection.[qv: 17a] Cells then adhere to the endothelial wall, which is guided by selectins, integrins and other adhesion molecules on the endothelium. After forming close adhesion, they move through the endothelial walls either by paracellular or transcellular transmigration to the inflamed sites, where they target resident cells and regulate the microenvironment. In response to certain stimuli, alterations or challenges under physiological or pathological conditions, the activated cells transfer multiple factors to navigate cells through the autocrine, paracrine, and telecrine pathways, ultimately regulating the inflammatory environment (**Figure** **6**). Since the recruitment of inflammation‐related cells plays a crucial role in the development and progression of various complex inflammatory diseases, understanding the mechanisms of these cells moving toward the targeted inflammatory sites is essential for designing cell membrane‐based nanotherapeutics to manipulate and regulate inflamed sites (**Table** **1**).

**Table 1:** Summary of inflammatory‐related cells and major receptors during recruitment

Cell	Functions	Priming receptors	Recruiting receptors
Neutrophils	Present antigen to T‐cell; immune cells activation; tissue and cartilage damage; generate toxic molecules to rapidly kill phagocytosed pathogens	CXCR1, CXCR2, PAFR, BLT1	L‐selectin, VLA‐4, LFA‐1, Mac‐1, α4β7 integrin, PSGL‐1
Macrophages	Act as sentinels through phagocytosis; antigen processing and presentation; destruction and clearance of microorganisms and apoptotic cells	CCR2, CCR6, CCR7, CCR8, CXCR1, CXCR2, CXCR4	L‐selectin, PSGL‐1, LFA‐1, VLA‐4
Lymphocytes	Antibody production; regulation of the immune response; cell‐mediated killing of infected or tumor cells	CXCR3, CXCR5, CCR1, CCR4, CCR5, CCR6, CCR7, CCR10, CX3CR1	LFA‐1, L‐selectin, CLA, α4β7 integrin, PSGL‐1
Platelets	Preserve vascular and tissue integrity; hemostasis and thrombosis; recruit immune cells to the inflamed sites; activate or adhere cells in the inflamed sites; pathogen clearance; tumor growth, metastasis and malignancy	LLR family, TLR family	GP IIb‐IIIa complex, αIIbβ3 integrin, PSGL‐1, GPVI
Stem Cells (MSCs)	Cell differentiation; regulate remodeling; tissue repair and regeneration; powerful immune response; trafficking to injured tissues	CXCR1, CXCR2, CCR1, CCR2, CXCR4, CX3CR‐1, CCR7, PDGF‐receptor, IGF1‐receptor	integrins (e.g., β1, β2, α1, α2, α3, α4, α5, α6, and αV), VLA‐4, P‐selectin, E‐selectin

*VLA‐4, very late antigen 4; LFA‐1, lymphocyte function‐associated antigen 1; Mac‐1, macrophage‐1 antigen; CCR, C‐C chemokine receptor; CXCR, C‐X‐C chemokine receptor; PSGL‐1, P‐selectin glycoprotein ligand‐1; CLA, cutaneous lymphocyte antigen; CX3CR, C‐X‐3‐C motif chemokine receptor; GP, glycoproteins; LLR, leucine‐rich repeat; TLR, toll‐like receptors; PDGF, platelet‐derived growth factor; IGF1, insulin‐like growth factor 1; PAFR, platelet‐activating receptor; BLT1, Leukotriene B4 receptor‐1.

#### 3.1.1 Neutrophils

Neutrophils, a crucial granulocyte member of the innate immune cell family, are considered the most abundant blood‐borne leukocytes (≈40–75%) in the healthy human body.^71^ These cells provide the initial barrier of the immune defense with its specialized granules.^72^ During inflammation, a tenfold increase in the production rate of neutrophils from the bone marrow in response to the inflamed site has been observed, indicating the strong affinity for and response to the inflammatory signals.^73^ Extensive evidence has highlighted that neutrophils contribute to the pathogenesis of human inflammatory diseases such as sickle cell disease, chronic obstructive pulmonary disease, Behcet's disease, ischemic stroke, acute pancreatitis, and inflammatory arthritis.^74^ Based on its relationship with these chronic diseases, neutrophils have been widely employed as targeting cells or carriers for cell therapies.^71^ Its targeting properties mainly depend on the phenotypic changes and surface molecules of neutrophils after leaving the bone marrow. For instance, “fresh” neutrophils released from bone marrow express CD62L and CXCR2, while “aged” or activated neutrophils in circulation display the phenotype CD62L^low^CXCR4^hi^CD11b^hi^CD49d^hi^ (CD11b and CD49d are the alpha subunits for integrin Mac‐1 and VLA‐4, respectively).[qv: 74b] The recruitment of neutrophils during inflammation undergoes a two‐step migration process, requiring cell‐molecular interaction and the interaction with activated endothelial cells. Given the great number of chemoattractant receptors sensing various signals (e.g., distress signals, pathogen‐derived or pathogen‐induced molecules) and adhesion molecules for inflammation‐induced counterreceptor binding, resting neutrophils could rapidly be activated and accumulate in the infection or inflammation site and play a predominant role during the initial stage of the inflammatory response. The adhesion process requires the conformational changes of integrin adhesion receptors such as VLA‐4 (α_4_β_1_ integrin), LFA‐1 (α_L_β_2_ integrin), and Mac‐1 (α_M_β2 integrin), assisting in the migration and locomotion of neutrophils to inflamed tissue.[qv: 74b,75] For example, LFA‐1 is involved in the adhesion of natural killer cells and cytotoxic T cells to their targets. Mac‐1 expression increased in neutrophils after stimulation, but this increase could hardly be seen in patients with glycoprotein deficiency syndrome. The activation of Mac‐1 contributes to a large aggregation of sickle red blood cells, inducing acute transient or prolonged obstruction of vascular blood flow. Acute inflammation such as myocardial infarction, stroke, and ischemia‐reperfusion requires neutrophils as key effector cells involved in the trafficking of molecules such as L‐selectin, PSGL‐1, LFA‐1, and Mac‐1. With its majority number in peripheral blood and irreplaceable role in innate immune responses as well as the pathogenesis of inflammatory diseases, neutrophils have become an ideal cell membrane source for cell membrane‐based nanotherapeutics targeting inflammation.

#### 3.1.2 Macrophages

Macrophages are highly versatile large white blood cells that work as major cellular effectors in inflammation and tissue repair processes. They differentiate from circulating peripheral‐blood mononuclear cells and migrate into tissues as long‐lived tissue‐specific macrophages or respond to inflammation.^76^ Macrophages participate in both innate and adaptive immune responses, where they act as sentinels responding to damage mainly through several functions, including phagocytosis, destruction, and clearance of microorganisms and apoptotic cells, and antigen processing and presentation.[qv: 76a] During diseases such as rheumatoid arthritis, Crohn's disease, multiple sclerosis, and atherosclerosis, macrophages are given distinct functional phenotypes due to various tissue types and environmental cues, from proinflammatory to anti‐inflammatory with remarkable plasticity.[qv: 76a] Based on its multifunctional properties, macrophages have been widely used to regulate pathogen recognition, inflammatory diseases, and tumor growth.[qv: 76b,77] With the help of chemokine receptors on the membrane surface (e.g., CCR2, CCR7, CXCR1),^78^ macrophages rapidly respond to pathogen‐associated signals, resulting in a sharp increase in trafficking to the inflammatory sites. Similar to other leukocytes, adhesion molecules such as PSGL‐1 (interacts with P‐selectin and E‐selectin) and L‐selectin (interacts with PNAd) from the macrophage membrane assist in the recruitment and adhesion of cells.^78^ In addition, transformed or pretransformed macrophages can regulate the process and fate of inflammation mainly through their properties of clearance and antigen presentation. Other receptor families, such as scavengers (phagocytosis, adhesion, etc.), GPI‐anchored receptors (Toll‐like receptors (TLR) signaling, apoptotic cell recognition, etc.), Ig superfamily (antibody‐dependent binding, uptake, killing, and regulation of inflammation), and TLRs (response to lipopolysaccharide (LPS) and peptidoglycan) are key effectors in clearance.[qv: 76a,79] In addition, as antigen‐presenting cells, processing and presenting antigens via interaction between MHC class II and T cells help macrophages participate in inflammatory regulation.[qv: 17a,76a,79] The phenotype of macrophages is rather plastic (M1 and M2 types), offering either proinflammatory or anti‐inflammatory functions with its surface receptors.[qv: 76a] The heterogeneity and plasticity in function provide more opportunities for engineering macrophages via dynamic environmental signals, thus supporting macrophages as major players in engineered cell membrane‐based nanotherapeutics by monitoring and orchestrating their phenotype and polarization status to regulate the inflammatory pathway while achieving the successful resolution of inflammation.

#### 3.1.3 Lymphocytes

Lymphocytes, including T, B, and natural killer (NK) cells, are a crucial subgroup of white blood cells with similar appearance but different functions. Different from NK cells (innate immune lymphocytes), T, and B cells, with their recombined antigen receptors, are important effectors of adaptive immunity.^80^ This relies on their functions of antibody production, cell‐mediated killing of infected or tumor cells, and regulation of the immune response. Thus, diseases such as pathogen infections, lung diseases (e.g., asthma, allergy, chronic obstructive pulmonary disease and tuberculosis), blood cancer, and other immune dysfunctions require lymphocyte participation.^81^ Lymphocytes are intrinsically mobile and continuously recirculating in a large cluster between the blood and the lymph in “high endothelial venules” (HEVs).^82^ Under inflammatory conditions, the HEV‐like structure can be observed in chronic inflammatory sites; thus, lymphocytes are triggered and migrate to inflamed sites.^83^ Its homing process largely requires G‐protein coupled receptors (GPCRs) responding to chemokines (CC, CXC, C, and CX3C ligand families) as well as TCR and costimulatory molecules.[qv: 82b,84] T lymphocyte migration in response to GPCRs has been thoroughly studied to understand the mode of lymphocyte migration.[qv: 84b] For example, Th1 cells express more CXCR3, CXCR6, and CCR5 in response to interferon (IFN) γ‐induced inflammation; CCR3, CCR4, and CCR8 are highly expressed on Th2 cells in response to interleukin (IL)‐4, 5, and 13. Naïve T cells can increase CXCR3 and CCR5 expression while downregulating CCR7, allowing them to effectively move from lymph nodes to inflammatory sites where activated chemokines are expressed (CXCR3: IP‐10, CXCL9, and ITAC; CCR5: RANTES, MIP‐1α, and MIP‐1β).^84^ In addition, “homing receptors” are also involved in migration and are specialized to engage respective tissue‐specific ligands. The combination of chemokine receptors and homing receptors provide a unique command code to determine the destination of lymphocytes. Therefore, T and B lymphocytes as well as other T cell subpopulations show different preferences in adhering to HEV sites. For instance, tissue‐specific homing receptors such as L‐selectin are for homing to lymph nodes, α4β7 integrin is for gut‐associated lymphoid tissue (GALT) and the gastrointestinal (GI) tract, and cutaneous lymphocyte antigen (CLA) is for skin.^85^ The selectivity in the principal homing receptor‐mediated homing process results from the permanent expression of their ligands at HEV sites (e.g., L‐selectin/CCL21, α4β7/MAdCAM‐1, CLA/E‐selectin).[qv: 84b,85] Although the mechanisms of lymphocyte receptors and their subsets have not been fully revealed, the complex and multiple regulations between chemokine and lymphocyte receptor expression give them various possibilities in targeting respective microenvironments during inflammation.

#### 3.1.4 Platelets

Platelets, derived from megakaryocytes via endomitosis, are small anucleate cells circulating in the blood stream. Beyond hemostasis and thrombosis, platelets have emerged as versatile effectors of the inflammation and immune response with specialized roles in host and adaptive defense to injury, which contributes to injury repair and resolution via recruiting inflammatory cells.^86^ Platelet activation is a common feature in the development of inflammatory diseases, including cardiovascular pathologies (e.g., unstable angina, acute myocardial infarction, and stent thrombosis), sepsis, inflammatory bowel disease, arthritis, or tumor.^87^ Recent studies have shown that platelet‐based therapy might be an effective approach for treating sepsis due to the importance of thrombocytopenia and the inflammatory cascades resulting from platelet activation during the development of disease.^88^ With their ability to activate microvascular endothelial cells and recruit immune cells to inflamed sites, platelets have been considered a new player in the mucosal scenario of inflammatory bowel disease. This multitude of clinical potentials stems from a unique set of platelet surface mediators correlated with immune evasion, subendothelium adhesion, and pathogen interactions. Resting platelets in the circulatory system are mostly activated by pathogen‐associated molecular patterns via TLRs, leading to cytokine release (e.g., platelet factor 4, RANTES or CCL5), which promotes the recruitment of circulating inflammatory cells.[qv: 86b] Like leukocytes, activated platelets then roll along and adhere to inflamed endothelium while interacting with other immune cells (mostly via P‐selectin). Membrane glycoprotein (GP) α^IIb^β_3_ (CD41/CD61) is the most abundant platelet adhesion receptor, transforming from a low‐affinity to a high‐affinity state for binding to fibrinogen, fibrin, von Willebrand Factor (vWF), and fibronectin vitronectin and promoting platelet aggregation after platelet activation.^89^ Both in vitro and in vivo studies suggest that there is a strong interaction between platelets and inflamed endothelium as well as other immune cells, which is mainly mediated by both platelet glycoproteins (GP) Ibα and α^IIb^β_3_.^90^ As an inflammatory inducer, platelets can interact with other inflammatory‐related factors through protein–protein interactions by LLR motifs. The GPIb‐IX‐V complex from the LLR family is the second most abundant platelet receptor, initiating and propagating hemostasis and thrombosis.^91^ In addition to direct interaction with pathogens, platelets can also adhere or activate various targeting cells in the inflammatory microenvironment. This process requires the translocation of P‐selectin from α‐granules to the platelet plasma membrane. In addition, endothelial cells and leukocytes are prime targets for platelets. Leukocytes tether to platelets via the PSGL‐1/P‐selectin interaction, among which monocytes adhere in a Mac‐1‐dependent (CD11b/CD18, αMβ2) manner and neutrophils interact with platelets through the surface TREM‐1/TREM‐1 ligand.^92^ Thrombin‐stimulated platelets particularly bind to macrophages rather than neutrophils, accelerating the processes of inflammation and blood coagulation.^93^ Other receptors such as C1q receptors (C1qR) can modulate platelet interactions with collagen and immune complexes at the site of vascular injury, inflammation and atherosclerotic plaques to tighten the connection.^94^ The ease of collecting platelet membranes can largely promote their application as drug‐loading vehicles and nanoparticle surface coatings, showing great benefits in prolonging circulating time and site targeting.

#### 3.1.5 Stem Cells

Stem and progenitor cell populations exhibit substantial contributions to tissue repair and regeneration through paracrine effects, regulating remodeling, differentiation, and, especially, the ability to traffic to injured tissues.^95^ Among these cells, MSCs, derived from perivascular cells (pericytes), can be widely found in adults, including in the bone marrow, adipose tissue, peripheral blood, lung, brain, and skeletal muscle.[qv: 95b,96] In addition to its self‐renewal and differentiation potential, MSCs are important immunoregulator cells in the body. Several in vivo disease models such as inflammatory bowel disease, arthritis, allergic encephalitis, and respiratory diseases have demonstrated the potent immune modulation properties of MSCs (summarized in review^97^). In most primordial conditions, MSCs can secrete a group of molecules to regulate the over‐zealous immune system as one of the first lines of defense against chronic autoimmune reactions and are thus considered guardians of inflammation. In response to inflammation, MSCs express a variety of chemokine and cytokine receptors (CXCR1, CXCR2, CCR1, CCR2, etc.) that can drive MSCs to the inflammatory sites by migrating toward inflammation and thus control inflammation locally.^98^ Some key receptor/chemokine pairs include CXCR4/SDF‐1, CX3CR‐1/fractalkine, CCR7/CCL21, as well as RANTES and MDC with their receptors (CCR2, CCR3, and CCR4).^99^ Overexpressing homing receptors such as CCR1 and CXCR4 could improve MSC migratory behavior toward injured and inflamed sites and the cancer microenvironment. Various researchers engineered MSCs to enhance their proliferation and functions by modifying several molecules.^100^ Among them, CXCR4 has been widely used for enhancing homing and migration properties for myocardial infarction repair and bone‐related diseases.^99^ Similar to leukocytes, MSCs undergo consecutive steps, including tethering, rolling, adhesion, extravasation, and engraftment, to achieve a successful homing process (comparison summarized in review^99^). A broad spectrum of integrin members including β1, β2, α1, α2, α3, α4, α5, α6, and αV are expressed on MSCs. Subunit‐like β1 is important for rolling and the firm adhesion of MSCs by interacting with vascular cell adhesion protein (VCAM)‐1 and extracellular matrix (ECM). Blocking β1 integrin on MSCs also reduced their engraftment in ischemic myocardium.^101^ To date, few studies have systemically and deeply explored the mechanism of stem cell migration to inflamed sites. At the site of inflammation, MSCs carry out their immunomodulatory actions by direct or indirect interactions with T cells, B cells, dendritic cells, and natural killer cells to regulate their functions in the localized tissue environment, which affect both innate and adaptive immune responses.^102^ The benefits of applying MSCs to cell‐based therapy and its potential for various engineering methods are summarized in a recently published review.^103^ Progenitor cells such as MSCs, along with their salient features in immunomodulation and low immunogenicity, are a competitive and powerful therapeutic membrane modality to be applied in advanced nanotherapeutics.

### 3.2 Cell Membrane‐Based Natural Detoxification Platform for Ameliorating Inflammation

Directly leveraging the entire cell membrane as a coating material makes it possible to preserve many of the properties exhibited by the source cell on the nanoparticles. This top‐down strategy offers numerous therapeutic opportunities from cell‐mimicking to multifaceted biointerfacing properties. By using the key properties of cell membranes, researchers have developed various formulations of cell membrane‐coated nanoparticles to selectively target the inflamed sites and regulate the microenvironment, mainly relying on their innate homing properties and specific membrane proteins.[qv: 74c,d,104] Among those researchers, Zhang's group has pioneered its innovative applications in drug delivery, imaging and photoactivatable therapy, detoxification, and immune modulation.^19^ In addition to simply applying its structural profile to enhance circulation time and target‐site accumulation, more attention has recently been placed on the intrinsic properties of disease‐related membrane surface receptors. With its conserved affinity for binding pathogenetic agents, cell membrane‐coated nanoparticles have been applied as decoys of susceptible cells to intercept and neutralize pathologic agents. Here, red blood cells were first introduced to fabricate nanosponges due to their high affinity for bacterial pore‐forming toxins, pathological autoantibodies, and nerve agents.[qv: 63b,105] Inflammatory‐related cells, such as neutrophils and macrophages, were later used to neutralize endotoxins and proinflammatory cytokines, resulting in the attenuation of sepsis and inflammatory arthritis, respectively. Until now, the concept of cell membrane‐coated nanoparticles as detoxification platforms has been applied in several different formats and conditions. Here, we chose four representative studies to highlight such a detoxification platform.

#### 3.2.1 Toxin Challenge

**Figure 7:**
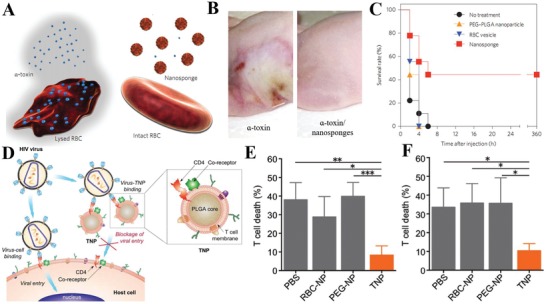
Cell membrane‐based detoxification for ameliorating inflammation. A) Pore‐forming toxins (PFTs) can be arrested and locked by red blood cell membrane‐coated nanosponges. B) Skin lesions on mice occurred 3 d following injection of α‐toxin or α‐toxin/nanosponges (*n* = 6 for each group). C) Survival rates of mice over 15 d after a tail vein injection of α‐toxin or α‐toxin/nanosponges (80 mg kg^−1^ nanosponges were injected 2 min after the toxin injection, *n* = 9). Reproduced with permission.[qv: 105b] Copyright 2013, Nature Publishing Group. D) Schematic representation of T‐cell membrane‐coated nanoparticles (TNPs) for attenuating HIV infectivity. CD4^+^ T cell membrane with CD4 receptor and CCR5 or CXCR4 coreceptors were used for viral targeting. TNPs neutralization against bystander T‐cell killing induced E) by gp120_IIB_ or F) with gp120_BaL_. Data shown as the mean ± SD, *p < 0.05, **p < 0.01, and ***p < 0.001. Reproduced with permission.^106^ Copyright 2018, Wiley.

Bacterial pathogenesis often results in life‐threatening infections in humans. One of its major routes is through the release of hemolytic toxins by gram‐positive bacteria, called pore‐forming toxins (PFTs), into the blood during infection. These toxins secreted by bacteria can spontaneously fuse with the cell membrane and form a heptameric structure that leads to uncontrolled permeation and cell lysis. Current detoxification treatment targets the molecular structure of toxins but is rather customized for different conditions. Since RBCs are preferred targets for toxins, a biomimetic toxin nanosponge functioning as a broad toxin decoy was designed (**Figure** **7**A–C).[qv: 105b] The obtained nanosponge was a typical core‐shell structure consisting of a PLGA core and RBC membrane coating (≈85 nm in diameter). The RBC membrane retained its toxin affinity after cloaking, while the inner polymeric core stabilized the nanosponge and prolonged systemic circulation. The nanosponges successfully protected RBCs from hemolysis caused by various PFTs (α‐toxin, streptolysin‐O, and melittin) and diverted them away from their cellular targets. In a mouse model, nanosponges significantly neutralized α‐toxin and improved the survival rate, especially for the nanosponge preinoculation group (11% mortality rate). This bioinspired RBC‐coated nanosponge shows the potential to absorb a wide range of PFTs regardless of their structure and thus may become a unique strategy for biodetoxification.

#### 3.2.2 HIV Infection

Human immunodeficiency virus (HIV) infection remains incurable, and depletion of immune cells (especially CD4^+^ T helper cells) is the hallmark of HIV infection, leading to acquired immunodeficiency syndrome (AIDS) and side effects. Although current antiretroviral therapy can keep plasma virus at an undetectable level, residual viruses in latent cells are the major obstacle for viral eradication. Virus entry begins with the interaction between viral envelope glycoproteins (e.g., gp120) and cluster of differentiation 4 (CD4) receptors, followed by binding to CCR5 or CXCR4 coreceptors on T cells. Inspired by their previous works on toxin detoxification, CD4^+^ T cells were collected as a plasma membrane source for the surface coating. The resulting T‐cell membrane‐coated nanoparticles (TNPs) preserved intrinsic surface markers critical for HIV binding, including human CD4 receptor and CCR5 or CXCR4 coreceptor with native conformation on the surface (Figure 7D–F).^106^ The PLGA core intimately interfaced with the membrane to avoid fusion and tailored the size of the TNPs. The results confirmed the selective binding of TNPs with gp120. By leveraging the natural affinity to cytopathic gp120, TNPs acted as T cell decoys to prevent the depletion of susceptible CD4^+^ T cells. This biomimetic decoy strategy enlarges host cell functions for viral neutralization without eliciting high selective pressure and has the potential to overcome the limitation of current antiretroviral therapy. While the in vitro results are promising, there is still a need to further optimize the pharmacokinetic profile and viral binding efficiency of TNPs for maximum in vivo outcome. Overall, this attractive work provides a potential therapeutic strategy to apply the affinity receptors from the source cell membrane to attenuate viral infection in general.

#### 3.2.3 Sepsis

**Figure 8:**
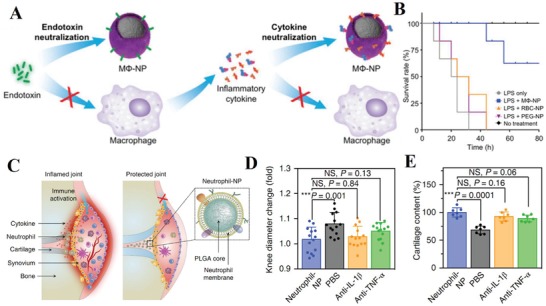
Cell membrane‐based detoxification for ameliorating inflammation. A) Macrophage membrane‐coated nanoparticles (MΦ‐NPs) as a two‐step process for sepsis management to neutralize endotoxins and proinflammatory cytokines. B) Survival rates of mice treated with LPS alone or LPS mixed with MΦ‐NPs, RBC‐NPs, or PEG‐NPs (*n* = 10). Reproduced with permission.^107^ Copyright 2017, National Academy of Sciences. C) Schematic illustration of neutrophil‐NPs designed for suppressing synovial inflammation and ameliorating joint destruction in inflammatory arthritis. D) Change of hind knee diameter of neutrophil‐NP‐treated mice on day 60 after arthritis induction compared to that on day 0. E) Cartilage content quantified from safranin‐O‐stained sections of mice in different treatment groups (*n* = 7; mean ± SD). Reproduced with permission.^108^ Copyright 2018, Nature Publishing Group.

Sepsis is characterized by an uncontrolled systemic inflammatory response to bacterial infections, leading to multiple organ dysfunction or failure. Endotoxin secreted by gram‐negative bacteria is an important pathogenic trigger upon its circulating in the blood stream and causes severe inflammatory responses and immune dysregulation. The neutralization and elimination of endotoxin is crucial for sepsis management; however, there is a challenge to find broad‐spectrum detoxification method with less clinical limits and toxicity. Since LPS is widely known as a pathogen‐associated molecular pattern (PAMP) by sentinel immune cells, it is possible to use its target cells such as monocytes and macrophage cell membranes as decoys. Recently, a biodegradable PLGA core coated with macrophage membrane (MΦ‐NPs) was developed for sepsis control (**Figure** **8**A,B).^107^ MΦ‐NPs possess an antigenic exterior identical to the source cell, inheriting their capability to bind to LPS via its LPS‐binding protein. Cognate pattern recognition receptors (PRRs) such as TLR4, CD14, and cytokine receptors (e.g., CD126, CD120a/b, and CD119) on the surface of macrophages were crucial to remove inflammatory cytokines (IL‐6, TNF‐α, and IFN‐γ). This protective efficacy was not observed for cells treated with RBC‐NPs or PEGylated nanoparticles under LPS conditions, demonstrating the unique property of macrophage membranes for LPS neutralization. Similar results were found in vivo, as MΦ‐NPs inhibited bacterial dissemination and subsequently improved survival rate while RBC‐NPs and PEG‐NPs failed. In theory, MΦ‐NPs can also be used in gram‐positive bacterial sepsis pathogens (scavenging lipoteichoic acids and peptidoglycan via TLR2/6) or fungal sepsis pathogens (β‐glucans via PRR Dectin‐1). The promising macrophage‐like nanoparticle demonstrates the potential of sepsis management via endotoxin neutralization and inflammatory cytokine sequestration, thus providing effective intervention for uncontrolled immune activation.

#### 3.2.4 Inflammatory Arthritis

Inflammatory arthritis, in terms of joint inflammation and damage, can result in devastating autoimmune diseases such as rheumatoid arthritis. Although precise causes remain unknown, the influx of immune cells and pathological molecules into the synovial joint space are prevalent during disease progression. These cells, originating from monocytes, macrophages, dendritic cells and T cells, together orchestrate the process of inflammatory arthritis. Due to the multiplicity of cytokine targets, none of the current treatments can act as broad‐spectrum anti‐inflammatory agents for inflammatory arthritis management. Inspired by their previous work using the cell membrane (RBC and macrophage, discussed in Section 3.2.1) as decoys to absorb and neutralize pathological molecules, the same group recently used neutrophil membrane‐cloaked PLGA (neutrophil‐NPs) to mimic neutrophils for inflamed joint targeting and immunoregulatory molecule binding (Figure 8C–E).^108^ The results showed that neutrophil‐NPs retained some key binding membrane receptors, such as TNF‐αR, IL‐1R, and LFA‐1, which would participate in cytokine binding and homing to activated chondrocytes and endothelium. Direct use of the membrane of effector cells of inflammatory arthritis gave neutrophil‐NP disease‐relevant functions, including neutralization of IL‐1β and TNF‐α, suppression of synovial inflammation, targeting the cartilage matrix, and providing chondroprotection against damage. Given the pivotal role of neutrophils in the inflammatory process, it appears logical to adopt neutrophil‐NPs in a wide range of inflammatory diseases.

### 3.3 Cell Membrane‐Based Nanotherapeutics Targeting Inflammatory Diseases

#### 3.3.1 Atherosclerosis

**Figure 9:**
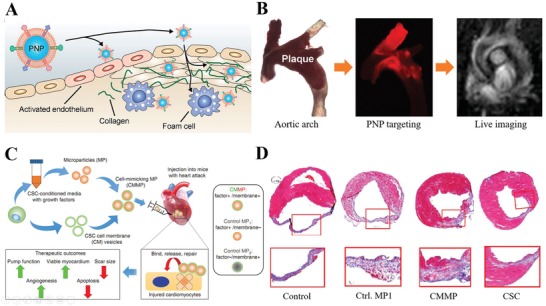
Cell membrane‐based nanotherapeutics targeting cardiovascular diseases. A) Schematic representation of platelet membrane‐coated nanoparticle (PNP) targeting different components of atherosclerotic plaques, including activated endothelium, foam cells, and collagen. B) In vivo targeting and live imaging of aortic arch from ApoE KO mice confirmed the presence of atherosclerotic plaques. Reproduced with permission.^109^ Copyright 2017, American Chemical Society. C) Schematic illustration of the biochemical design and study model of CMMPs. MPs were fabricated from PLGA and conditioned media of human CSCs, and then MPs were coated with membrane fragments of CSCs to form CMMPs. D) Masson's trichrome‐stained myocardial sections 4 weeks after treatment with Control PBS, Control MP1, CMMPs, or CSCs. Reproduced with permission.^110^ Copyright 2017, Nature Publishing Group.

Atherosclerosis plays an important role during the development of the most common and fatal cardiovascular diseases (e.g., coronary artery disease and cerebrovascular disease). This initiates with the dysfunction of the endothelial layer and promotion of local inflammatory as well as circulating cell recruitment, thus resulting in arterial plaque, collagen‐rich fibrous cap development, plaque rupture, or even heart stroke. Early detection would be helpful, but patients are usually asymptomatic until the very late stage. Based on the mechanisms during atherogenesis, cells such as monocytes and platelets can be considered potential candidates for targeting atherosclerotic sites. Recently, one proof‐of‐concept work on MRI of atherosclerotic sites leveraged platelet membranes to design a biomimetic nanoparticle‐based imaging platform (**Figure** **9**A,B).^109^ Here, a platelet membrane was directly coated on a PLGA core to form a thin surface layer, with its zeta potential close to platelet vesicles. Compared with bare PLGA core or RBC membrane‐coated nanoparticle, the platelet membrane guided the nanoparticle to bind various components of atherosclerotic plaques (e.g., foam cells, collagen, activated endothelium, and plaque). This targeting ability was also confirmed by an aortic arch sample from an atherosclerosis mouse model, showing effective localization of nanoparticles and their payload release at the plaque region and preatherosclerotic sites. The nanoparticle inserted with the MRI contrast agent also generated clearer results to distinguish the plaque sites for live imaging and provided information about the underlying biology of the plaque site. As shown in the pioneering work here, this platelet‐coated nanoparticle, with its effective localization to atherosclerotic sites, can become a novel live imaging tool to better assess and manage the development of atherosclerosis. It is possible to use cell membranes to detect the development of diseases such as atherosclerosis and further probe for potentially diseased tissue at an early stage.

#### 3.3.2 Inflamed Vasculature Disease

Blood vessels are essential in the regulation of various functions such as the expression and release of proteins and factors, tissue fluid homeostasis, selective transport, and immune response. As the key component of the vasculature, endothelium dysfunction has long been associated with diseases such as autoimmune disease, acute lung inflammation, atherosclerosis, sepsis, and cancer. However, this biological barrier also limits the optimal biodistribution and tissue‐specific targeting of drugs, resulting in low therapeutic efficiency. Inflamed/activated vasculature can activate the recruitment of immune cells in the circulating blood stream, including platelets, leukocytes, lymphocytes and some stem cells. Based on the close connection between activated endothelium and recruited cells, enriched proteins from leukocytes' plasmalemma were incorporated into lipid nanoparticles (Figure 3D,E).[qv: 30b] The resulting proteolipid vesicles, here regarded as leukosomes, inherited the versatility and physicochemical properties of leukocytes and liposomes (best protein–lipid ratio). Some key surface proteins (e.g., LFA‐1, Mac‐1, PSGL‐1, and CD47) related to leukocyte adhesion to inflamed endothelium and self‐tolerance were detected on leukosomes, therefore providing leukosomes with selective targeting of inflamed endothelium. In addition to enabling nanoparticles to evade the mononuclear system, leukocyte membrane proteins bestow leukosomes with a great capability of negotiation across various biological barriers and thus effectively alleviated inflammatory conditions in an LPS‐induced mouse model when loaded with dexamethasone. The combination of top‐down and bottom‐up strategies create chimaeric leukosomes as a new method to exert more advanced drug‐delivery platforms for the treatment of chronic inflammatory diseases.

#### 3.3.3 Ischemic Disease

Ischemia usually occurs when the blood supply is abruptly cut off, which is followed by irreversible tissue damage and is thus often associated with a severe inflammatory response and mortality. Recently, noninvasive cell therapy applying ischemic tissue‐directed homing response has generated positive results. Based on this mechanism, cells such as neutrophils,[qv: 74c] monocytes and their descendant macrophages, dendritic cells, lymphocytes, and stem cells would be potential cell membrane sources. As summarized in Table 1, inflammation‐directed migration of MSCs is mediated by CXCR4 on the plasma surface. Given this feature, one recent report studied the therapeutic potential of membrane‐coated nanoparticles in an ischemic hindlimb model by engineering human adipose‐derived stem cells (hASCs) to overexpress CXCR4 on the membrane as the coating source to encase the vascular endothelial growth factor (VEGF)‐loaded PLGA core.^109^ Nanoparticles with enriched CXCR4 receptor (BSMNCs) showed better accumulation in ischemic tissue. Together with loaded VEGF, blood reperfusion, muscle repair, and limb salvage were improved in the BSMNC group. Another study focusing on myocardial infarction (MI) designed a synthetic cell‐mimicking microparticle (CMMP) recapitulating cardiac stem cell (CSC) functions in tissue repair (Figure 9C,D).^110^ Here, they loaded the CSC secretome (mainly VEGF, IGF‐1, and HGF) inside the PLGA core and cloaked CSC membrane fragments on the surface. CMMPs could firmly bind with cardiomyocytes, and biointerfacing between CMMPs and cardiomyocytes was further revealed by time‐lapse imaging of CMMPs rolling and travelling on the attached cardiomyocytes. Similar to CSC, CMMPs showed the best preservation effects on myocardium and moderated scar formation in the MI model but did not stimulate T‐cell infiltration. The findings above highlight the promising potential for recapitulating the natural stem cell functions of paracrine effects and tissue homing for various ischemic diseases.

#### 3.3.4 Inflammatory Bowel Disease

**Figure 10:**
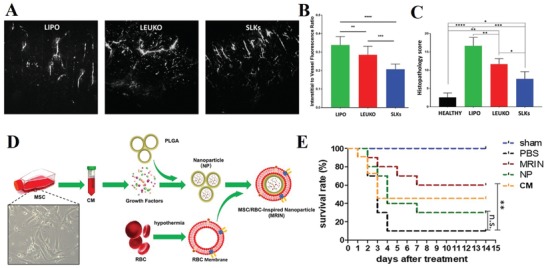
Cell membrane‐based nanotherapeutics targeting gastrointestinal diseases. A) Ex vivo imaging of colons from mice treated with liposomes (LIPO), leukosomes (LEUKO), or specialized leukosomes (SLKs). B) The relative fluorescence intensities between the outside and inside of the vessels (*n* = 10 vessels per image). C) Histopathological scores of colon tissues of healthy mice and DSS mice treated with different nanoparticles (*n* = 6). *p < 0.1; **p < 0.01; ***p < 0.001; ****p < 0.0001. Reproduced with permission.[qv: 30d] Copyright 2017, Royal Society of Chemistry. D) Schematic illustration showing the fabrication of MRINs by coating nanoparticle containing MSC‐conditioned media. E) Survival rates of animal after different treatment (*n* = 10 per group). Reproduced with permission.^111^ Copyright 2018, American Chemical Society.

Inflammatory bowel disease, including Crohn's disease and ulcerative colitis, is a systemic and chronic inflammatory condition of the GI tract. Although its general pathogenesis and etiology remain unclear, all causative factors can disturb intestinal homeostasis and result in a severe inflammatory cascade. Suppression of the inflammatory immune response via drugs or biologics can control the chronic inflammation in the bowel but may cause serious side effects due to typically high dosage required. Given the important role of immune cells, such as monocytes, neutrophils and lymphocytes, in regulating the immune response as discussed before, these cells have been used to design bioinspired nanoplatforms by transferring membrane proteins to synthetic carriers. Here, researchers designed leukosomes to explore their therapeutic potential by overexpressing α4β7 integrin on the leukocyte surface ex vivo and followed the same protocol to obtain 120–160 nm “specialized leukosomes” (SLKs) (**Figure** **10**A–C).[qv: 30d] Thus, SLKs can mimic T‐cell recruitment to the gastrointestinal tract, where α4β7 integrin is responsible for binding mucosal addressin cell adhesion molecule (MAdCAM)‐1 overexpressed on the endothelial membrane. Compared with liposomes and leukosomes (mainly in the interstitial space of colon), SLKs actively accumulated in the inflamed colon and were firmly bound with the activated endothelium in diseased colon tissue. Edema in colon tissue was largely reduced, and vascular function, both blood and lymphatic, were improved. The reduction in inflammation might be due to the “competitive binding” of SLKs to inflamed vasculature that blocks the adhesion and transmigration of immune cells. Self‐derived biological materials such as SLKs exhibit great potential for use in natural anti‐inflammatory nanotherapeutics and targeted drug delivery systems with enriched membrane proteins to enhance the repair process of specific tissues/conditions.

#### 3.3.5 Acute Liver Failure

Acute liver failure is an uncommon but lethal disease caused by insults such as viral infections and toxic reagents. It is characterized by global hepatocyte necrosis, rapid deterioration of liver function, coagulopathy and thus multiorgan failure. Recently, an alternative stem cell strategy using MSCs showed successful mitigation in animal models and human trials. To overcome its limit as a “live drug,” researchers fabricated cell‐mimicking nanoparticles encapsulating MSC‐secreted factors and explored its therapeutic potential for acute lung failure (Figure 10D,E).^111^ In this study, the RBC membrane was chosen to encase the PLGA core with the MSC secretome (e.g., HGF, SDF‐1, and IGF‐1), referred to as MRINs. RBC coating prolonged the blood retention of nanoparticles but did not influence the growth factor release. In the CCl4‐induced liver failure murine model, protection of liver functions with reduced proinflammatory cytokines and hepatic apoptosis, as well as higher levels of regeneration, were observed in the MRIN group. Compared with their cell‐mimicking MSC particles (MSC secretome+MSC membrane), nanosized MRIN (≈200 nm) were better retained in liver and had increased colloidal stability and bioavailability after intravenous delivery.

#### 3.3.6 Tumor Inflammatory Microenvironment

Inflammation is one of the classical hallmarks of cancer, where activated immune cells such as macrophages, neutrophils, and lymphocytes home to the tumor sites. The sustained presence of inflammatory mediators maintains the advantage of an environment suited for tumor cell proliferation. The extravasation of tumor cells through the stroma also requires the assistance of proinflammatory mediators. Pioneering studies on cell membrane‐based tumor targeting started from blood circulation and accumulation of nanoparticles to tumor sites. With simple membrane collection methods, RBC membrane‐camouflaged nanoparticles become an attractive candidate for various cancer therapies in the early stages. A recent study has also designed cancer cell membrane‐camouflaged nanoparticles for homogenetic targeting of tumor sites.^64^ We will summarize recent studies on the application of inflammatory‐related cell homing and targeting to tumor sites for cancer therapy.

**Figure 11:**
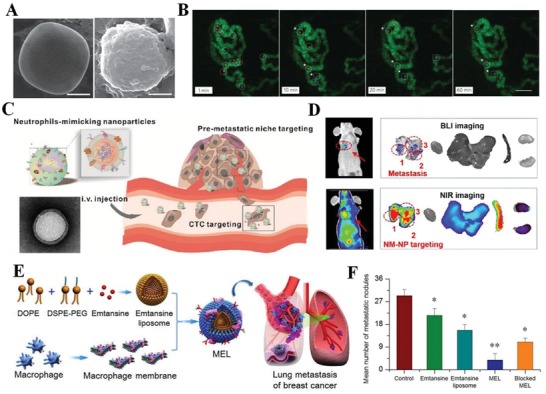
Cell membrane‐based nanotherapeutics for the inflammatory tumor environment. A) Representative SEM images of a bare NPS surface (left) and an NPS camouflaged with leukocyte‐derived membranes (right). Scale bars, 1 µm. B) Representative images of circulating and adherent LLVs (red, circles) and NPS (blue, squares) in the melanoma microvasculature (green) at different times (scale bar 100 µm). Reproduced with permission.[qv: 30c] Copyright 2013, Nature Publishing Group. C) Schematic representation of carfilzomib‐loaded NM‐NPs (NM‐NP‐CFZ) designed for neutralizing CTCs in circulation and preventing early metastasis. D) Dual‐mode imaging of NM‐NP‐CFZ‐treated mice with obvious metastasis 14 d after luc^+^ 4T1 cell injection in vivo and ex vivo. Reproduced with permission.^72^ Copyright 2017, American Chemical Society. E) Scheme of macrophage membrane‐coated emtansine liposomes (MELs) with specific targeting to suppress lung metastasis in breast cancer. F) The average number of macroscopic lung metastatic nodules from each treatment group, *p < 0.05, **p < 0.01. Reproduced with permission.^112^ Copyright 2016, American Chemical Society.

Most aggressive tumors start from carcinoma in situ before spreading to other sites. To achieve effective and site‐specific targeting, reduce side effects and enhance therapeutic efficacy, a drug delivery system must be able to negotiate its way past the vascular barrier and arrive at the tumor tissue in sufficient quantities. Given the complexity of mass transport in the blood stream, “biomimetic camouflage” systems, especially membrane‐based vehicles, are promising due to their natural interaction and communication with the surrounding environment. Since activated endothelial cells from the inflamed vasculature are responsible for recruiting cells to the tumor sites, it would be ideal to use cells such as leukocytes, platelets, and lymphocytes to camouflage the drug and deliver it to the tumor sites. A proof‐of‐concept was published in 2013 by coating nanoporous silicon (NPS) with leukocyte membranes, termed leukolike vectors (LLVs), to avoid opsonization and preferentially bind to inflamed endothelium for enhanced transport across the endothelium (**Figure** **11**A,B).[qv: 30c] The resulting LLVs retained critical molecules such as CD45, CD3z, Lck, and LFA‐1 from leukocytes, which were important during homing and targeting. Moreover, LLVs improved tumoritropic accumulation in vivo and thus achieved better results. These LLVs are a promising candidate for chemotherapeutic delivery to cancer cells.

In the severe stage of tumor development, metastasis contributes to over 90% of cancerous tumors and cancer‐related mortality. To form metastasis, the survival and dissemination of circulating tumor cells (CTCs) require the engagement of inflammatory cells, chemokines, and cytokines. Together, the colonization of CTCs and their soil, “(pre)metastatic niche,” become valuable diagnostic tools and therapeutic targets for metastasis treatment. Compared with other treatments, cell membrane‐guided targeting has revealed positive results. For example, camouflaged platelets have been used to target CTCs and their microthrombi in circulation and greatly prevented metastasis. Other inflammation‐related cells such as neutrophils and macrophages have also been applied to target metastasis due to their close interaction with the niche or tumor microenvironment. Neutrophil membrane‐coated PLGA (NM‐NP) exhibited much higher CTC‐capture efficiency in vivo and improved migration to the premetastatic niche (Figure 11C,D).^72^ When loaded with carfilzomib, NM‐NP selectively inhibited the progression of metastasis. In another study, a macrophage membrane was used to encase emtansine liposomes targeting lung metastatic sites of breast cancer (Figure 11E,F).^112^ In analyzing macrophage binding to metastatic cells via the α4β1 integrin‐VCAM 1 interaction, this study shows that macrophage decoration effectively improved metastatic targeting in the lung and therefore suppressed lung metastasis. This advanced strategy reserved the homing and targeting properties of cells while reducing the drawbacks such as short half‐life and potential risks after transplantation, providing a new method for cancer metastasis prevention and therapy.

## 4 Opportunities and Challenges for Clinical Translation

### 4.1 Translation Potential of Cell Membrane‐Based Therapy

Numerous studies have demonstrated a close correlation between inflammation and the pathogenesis of many unresolved inflammatory diseases. In these cases, inflammatory cells are recruited to the inflamed sites. This motivates the application of engineered cell membrane‐based nanotherapeutics to treat chronic or acute inflammatory diseases (summary from

**Figure 12:**
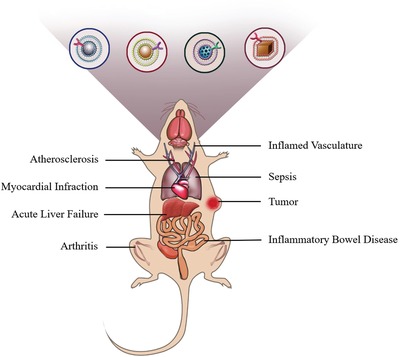
Schematic illustration of cell membrane‐coated nanoparticles treating inflammatory‐related diseases in a mouse model. Different cell membrane‐coated nanoparticles have been applied in various inflammatory‐related diseases, such as atherosclerosis, myocardial infarction, acute liver failure, arthritis, sepsis, inflamed vasculature, inflammatory bowel disease, and tumors, and have shown positive therapeutic results. See details in Section 3.

**Figure 13:**
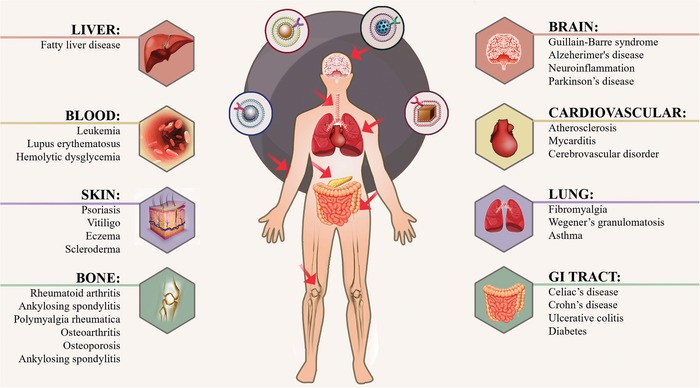
Schematic illustration of cell membrane‐coated nanoparticles to treating different human inflammatory‐related diseases. Different cell membrane‐coated nanoparticles can be applied in various inflammatory‐related diseases occurring in organs such as the brain, heart, lung, liver, and skin in the future.

Section 3, **Figure** **12**). For those diseases, the treatment might be more effective if it could be tailored to the patient's own conditions, and patient‐derived cells may be the best membrane source. Cells such as red blood cells, platelets, neutrophils, monocytes, and lymphocytes can be expanded ex vivo prior to membrane surface engineering. In some cases, certain types of cells are difficult to obtain directly, and somatic stem cells or induced pluripotent stem cells (iPSCs) can be used instead by directed differentiation into the desired cell types. Cell therapy has shown great promise in addressing unmet medical needs in the past decade. Hemocytes, e.g., red blood cells, have been widely used for autologous cellular therapy for decades, establishing their clinical utility.^113^ Recent clinical success of adoptive cell transfer (ACT) therapies, such as chimeric antigen receptor (CAR‐T) cells for cancer, have attracted more interest and driven the development of cell membrane‐based therapeutics. As discussed in Section 3, the membranes derived from well‐characterized inflammatory cells have been widely used as vehicles and show significant improvement in targeting and therapeutic efficacy in inflammation disease models, including arthritis, atherosclerosis, inflammatory bowel disease, pathogen clearance and cancer. These biomimetic systems can not only reduce nonspecific uptake but also increase specific targeting toward the disease site for drug delivery, imaging, and externally triggered therapy. Since many chronic diseases result from dysfunction and dysregulation of inflammatory conditions, inflammatory cell membrane‐based nanotherapeutics can be a promising personalized treatment in the future. Thus, other diseases such as Alzheimer's disease, Parkinson's disease, diabetes, asthma, ischemic brain injury, chronic obstructive pulmonary disease, multiple sclerosis, glioblastoma and autoimmune encephalomyelitis can all benefit from the aid of intelligently engineered, cell membrane‐based nanotherapeutics (**Figure** **13**).

### 4.2 Challenges toward Clinical Translation

Although inflammatory cell membranes hold promise in anti‐inflammation therapy, there are technical obstacles hindering the translation of cell membrane‐based nanotherapeutics. This field is still in its early stages as most studies have only been performed in small animals. To achieve clinical translation, the following considerations deserve attention:

1) *Maintaining relevant cell phenotypes in large‐scale ex vivo cell expansion*: Expansion may alter cell phenotypes. Most of the current ex vivo cell culture systems do not have any in situ monitoring of phenotypic changes or feedback regulation to tune the culture conditions. Particularly in large‐scale production, it would be difficult to control the purity and quality of the whole population. To obtain a functional membrane for coating, a standardized culture protocol without compromising the desired phenotype should be established and validated.^114^ For example, GMP standards for clinically used immune cells, such as dendritic cells (DCs) and T cells, have already been established for expanding patient cells and maintaining their phenotypes. For stem cells, methods with various 3D bioreactor designs have also been reported for large‐scale culture with better lineage control. All of these examples provide insight into the potential solution for maintaining the phenotype and quality of the cell resource.
2) *Achieving simple, effective, and reproducible membrane engineering*: To target the inflammatory microenvironment, surface engineering or modification of the cell membrane is attractive. Only a few of the methods (physical, synthetic and, genetic/nongenetic strategies) in Section 2 are designed for clinical use. Compared with lab‐scale protocols, production for clinical use may require more automation to realize precise, controllable, cost‐effective and reproducible handing of tailored cell membrane materials.^115^ The production of FDA‐approved immunotherapies, such as DC‐based vaccines and CAR‐T cells, suggests ways in which we could improve the current modification methods to meet clinical requirements.
3) *Improving the cell membrane extraction and coating procedures*: Membrane extraction and coating require a more rigorous protocol to ensure the product's uniformity, integration, cost, and reproducibility. A typical membrane collection and coating protocol includes cell lysis, sonication, centrifugation, and extrusion. Each step would cause some loss, and the membrane uniformity may sometimes be hard to control. To address these issues, an integrated, automated system would be attractive. It would serve as an integrated platform to allow cell membrane‐coating manufacturing, from membrane extraction to coating, all done on one device. In addition, the cell membrane alone cannot function as a drug carrier without a core nanoparticle, and it can also control the release of loaded drugs or reagents. As the focus of this review is cell membrane engineering, more details on core particle selection can be found in other specific reviews.
4) *Improving storage of cell membrane‐coated NPs*: With improvement in cell membrane collection and coating procedures, a robust storage system should also be developed to maintain the quality of engineered membrane‐coated nanoparticles. The storage conditions, including solvent type, particle concentration and temperature, would affect the shelf‐life of the nanoparticle itself or the coating. Although there is no conclusion regarding the best way for membrane‐coated nanoparticles to be stored, the storage methods for liposomal drugs would offer guidance to determine the best method because of the similarity between the liposomal and cell membrane systems.^116^ Most commercial liposome‐based drugs are lyophilized products, and they can be stored for up to 48 months. Similarly, storing the membrane‐coated nanoparticles in an aqueous solution may cause physical and chemical instabilities in long‐term storage. In contrast, several studies showed that freeze‐drying could increase the shelf‐life of cell membrane‐coated nanoparticles, and they can be later reconstituted with saline for administration.
5) *Standardizing the protocol and establishing proper quality controls for preclinical and clinical studies*: For clinical translation, standardization of the protocols and technology with proper quality controls for cell engineering and manufacturing would be required. Some quantitative evaluation criteria, such as biological safety, stability in solvent, dispersibility, biodistribution, and biotoxicity of the engineered cell membrane–coated nanoparticles, should be first and clearly characterized. Although several studies with preclinical settings have been conducted to validate this system, those criteria are extremely important to avoid raising any safety concerns in patients when it enters clinical trials, as this would be a new drug formulation.

### 4.3 Precise Manipulation of the Cell Membrane in Inflammation

The complexity, variability and heterogeneity of the patient‐specific inflammatory network and its corresponding cellular interactions result in great variation in the treatment response among patients. Thus, personalized nanotherapeutic solutions need to be versatile and adaptive to better ensure that such treatment is given only to the patients who would benefit from it. Compared with classical therapeutics, a 2D treatment system, considering both temporal and spatial aspects, should be established. A typical 2D approach includes specific targeting, real‐time monitoring of inflammatory microenvironment dynamics and controlled release of inflammation‐targeted drugs.^117^ As highlighted in this report, the homing capability of circulating cells to the inflammatory sites inspires the rapid development of targeted therapy, which provides a more effective and less toxic treatment option over conventional therapy. However, we should also evaluate the off‐targeting effect of the system to prevent undesired accumulation on other tissues, which may lead to drug resistance. Since the homeostasis and dysregulation of inflammation cannot occur without multiple interactions between cells and the microenvironment, there is a need for real‐time monitoring of the dynamics of these interactions to tune the nanotherapeutic dosage to maximize the therapeutic efficacy for different stages of diseases. Inspired by the newly reported idea that combines diagnostic and therapeutic components within a smart nanorobot, cell membrane‐based nanotherapeutics may also be designed to adjust the drug dose through feedback from the microenvironment in the future. In other words, a best‐matched cell membrane‐based nanotherapeutics in response to different microenvironment feedback and the stages of the disease can greatly improve the therapeutic efficacy. The field of engineered cell membrane‐based nanotherapeutics still has ample room for improvement, particularly by changing the dysregulated network back to the homeostatic stage. This requires a deep understanding of the interaction and communication between various inflammatory cells and their specific phenotypes and surface components. It may be fruitful to investigate the influence and/or application of nonimmune cell type membrane in inflammation‐targeting nanotherapeutics. The future of personalized nanotherapeutics alleviating suffering and preventing death caused by chronic or acute inflammatory diseases will hinge on our ability to confine inflammation at its cellular and molecular levels.

## 5 Conclusions

Cell membrane‐based nanotherapeutics, which integrate both the intrinsic properties of cells and the functional versatilities of nanomaterials to deliver therapeutics with tissue‐targeting precision, offer new therapeutic strategic options. Compared with other natural or synthetic vehicle systems, natural cell membranes hold targeting capability through the interactions between their surface components and those of the target cells, so we could transport and control the payload release at the inflamed site. Because of their unique properties, cell membranes have been applied in drug delivery (e.g., doxorubicin and paclitaxel), antibacterial infections, detoxification, autoimmune diseases, vaccine delivery and MRI for injury or cancer localization (summarized in refs. 19, 118). By translocating the entire cell membrane onto the surface of nanoparticles, the biological surface moieties may be preserved, including those for immune evasion and targeting. Furthermore, native cell membranes can be engineered to enhance the targeting and therapeutic potential the approach. Given the complexity, variability and heterogeneity of the patient‐specific inflammatory network and its corresponding cellular interactions, successful translation of cell membrane‐based nanotherapeutics into the clinic can be socioeconomically rewarding yet challenging. Future success lies in a better understanding of the inflammatory microenvironment and advanced techniques to precisely control these cell membrane‐based systems. There is still ample room for engineered cell membrane‐based nanotherapeutics to develop, as many groups are continuously working on refining the current methodology for formulating industry‐wide benchmarks and manufacturing principles. Convergence of nanotechnology, medicine, material science, bioengineering and pharmaceutical perspectives can help to reduce development costs, alleviate the gap between preclinical research and clinical translation, and ultimately bring more efficacious cell membrane‐based nanotherapeutics to patients.

## Conflict of Interest

The authors declare no conflict of interest.
